# Registration of Nurses

**Published:** 1891-03-07

**Authors:** 


### The Registration of Nurses.

We have been requested to publish the following resolutions unanimously adopted at a meeting of representatives of Hospitals and Nurses' Training Schools, held at St. Thomas's Hospital, on Friday, the 27th ult. :—

The resolutions were spoken to by the following gentlemen : Sir Rutherford Alcock, K.C.B., Vice-Chairman of the Westminster Hospital; Mr. W. Rathbone, M.P., as the representative of Miss Nightingale and the Nightingale Fund ; Dr. J. S. Bristowe ; Mr. E. H. Lushington, Treasurer of Guy's Hospital; Dr. Alchin, Westminster Hospital; Canon Troutbeck, Treasurer of the Westminster Hospital; the Rev. Dr. Wace, Chairman of the King's College Hospital and Training School; Dr. Steele, of Guy's Hospital, and others. It was unanimously resolved :—

That this Meeting of Representatives of Nurse Training Schools, and others, desires to enter its protest against the conferring by the Board of Trade upon the British Nurses' Association the privilege which it seeks, viz. : a licence to register without the word "*Limited*" to its name, in pursuance of the provisions of the 23rd section of the Companies' Act, 1867—

For the following reasons, viz. :—

1. That the main object of the British Nurses' Association is to "form, control, and carry on a Register of Trained Nurses, or to take over, control, and carry on, any such register as may have been, or shall hereafter be established by any person or persons, or association, whether incorporated or not" ; or, in other words, to establish and control a General Register of Nurses, which shall possess an authoritative character.

2. That a General Register, containing, as it ought, in order to be of any real service to the public, a complete statement of the essential qualification of each individual, is not adapted to the calling of a nurse, as from the nature of the case it could not be otherwise than imperfect and untrustworthy, and, therefore, misleading to the public and the medical profession.

3. That such a register would be prejudicial to the interest of the nurses themselves, by lowering the position of the most competent, without corresponding improvement to the less capable, and would therefore have the effect of lowering the standard of nursing generally.

4. That the British Nurses' Association being opposed in its views by all the leading Nurse Training Schools, and not having any substantial claim to represent the great body of nurses for the sick, is not entitled to claim the privilege which it seeks ; and that a copy of these resolutions, signed by the chairman of the meeting, be forwarded to the President of the Board of Trade.

It was further resolved that each of the large hospitals and nursing institutes of the country be requested to nominate two representatives to form a Committee of Observation, and that this committee, should it consider it necessary, be empowered to request theP resident of the Board of Trade to receive a deputation on the subject.

